# An Incidental Case of Sarcoidosis in a Young Male Patient Presenting With Symptomatic Chronic Cholecystitis

**DOI:** 10.7759/cureus.75183

**Published:** 2024-12-05

**Authors:** Kelsey A Clabby, Yelena Piazza, Vladimir Neychev

**Affiliations:** 1 Surgery, University of Central Florida College of Medicine, Orlando, USA; 2 Pathology, University of Central Florida College of Medicine, Orlando, USA

**Keywords:** cholethiasis, gastric sarcoidosis, hepatobillary surgery, incidental diagnosis, incidental surgical finding, laporoscopic cholecystectomy, noncaseating granuloma

## Abstract

Sarcoidosis is a multisystem disease process with a bimodal distribution typically affecting African American women and those of Scandinavian descent characterized by noncaseating granulomatous disease. We present a case of a 29-year-old African American male patient who was seen in the clinic for recurrent symptomatic cholelithiasis. He had no past medical history or symptoms besides intermittent postprandial right upper quadrant (RUQ) pain with imaging confirming cholelithiasis. He was taken for elective robotic cholecystectomy, and an intraoperative liver biopsy was performed due to incidentally found widespread miliary liver lesions and an enlarged pericystic lymph node. The pathology from the intraoperative sample showed noncaseating granulomas consistent with sarcoidosis, a rare presentation in both the liver and pericystic lymph node.

## Introduction

Sarcoidosis is a multisystem disease process that has a bimodal age distribution (early and late adulthood) commonly presenting in African American women and those of Scandinavian descent [1]. The incidence ranges between 2.3 and 11 cases per 100,000 with a prevalence between 2.17 and 160 per 100,000 individuals [2]. It presents with a noncaseating granulomatous disease that typically impacts the lungs and lymph nodes (90%-95%), where gastrointestinal (GI) and hepatic presentation are not as common and even less common in the gallbladder and associated lymph node [1]. The exact cause of sarcoidosis is unknown, although various theories point to immunological, genetic, and environmental factors [3]. The presenting symptoms can vary, ranging from general constitutional symptoms such as fatigue, fever, and weight loss to no symptoms at all [2]. Some of the most common presenting symptoms are from the lungs and upper respiratory tract, including cough, dyspnea, and chest pain. However, skin changes, fever, and arthritis are not uncommon as well [4]. In the majority of cases, however, the disease is indolent, and the diagnosis comes to light incidentally, following an imaging of the chest for other reasons [3]. In the cases of abdominal involvement, these findings are often not obvious and are frequently missed on CT scans [5].

Here, we describe a case of a young African American male patient who presented with the clinical and paraclinical picture of chronic calculous cholecystitis without clinical signs or symptoms of sarcoidosis. He was diagnosed with sarcoidosis after an intraoperative biopsy of incidentally found suspicious miliary liver lesions and an enlarged pericystic lymph node.

## Case presentation

**Figure 1:**
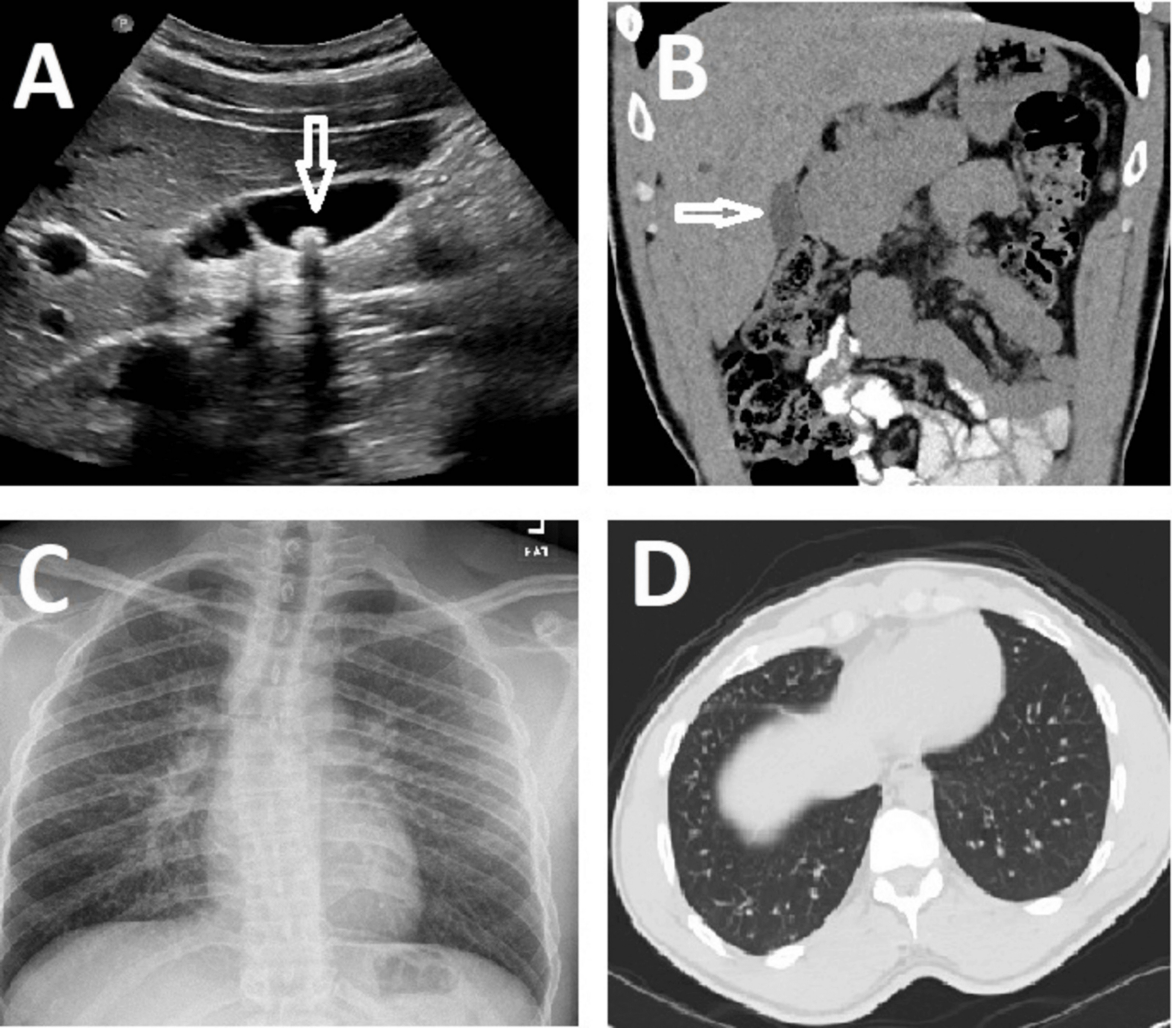
Multimodal Imaging Findings (A) Sagittal ultrasound image of the gallbladder representative of a hyperechoic gallstone with posterior acoustic shadowing (arrow). (B) Representative coronal CT image showing no evidence of acute cholecystitis or pericholecystic fluid (arrow). (C) CXR image with no overt pathologies. (D) Representative axial CT image showing mild airway thickening in the lung bases. CT: computed tomography, CXR: chest X-ray

The patient is a 29-year-old man who presented for evaluation following a recent emergency room (ER) visit for possible gallstone-related abdominal pain. He started experiencing similar symptoms one year prior when he was diagnosed with gallstones found on ultrasound. Since then, he has made lifestyle changes by decreasing red meat intake and increasing exercise, but he has had persistent abdominal pain over the following years. He felt the pain was well controlled until one week prior to the presentation when he had sudden severe epigastric and right upper quadrant (RUQ) abdominal pain, radiating to mid-back and right shoulder, which made him seek out treatment at the ER. He had an ultrasound that again showed gallstones with no wall thickening, pericholecystic fluid, and negative Murphy’s sign (Figure 1A). In the ER, he had a complete blood count (CBC) and comprehensive metabolic panel (CMP), which were unremarkable, and he was referred to general surgery for cholecystectomy.

The patient had no further relevant past medical history, surgical history, or family history. He endorsed smoking cigarettes daily with unclear amount and duration. He also endorsed drinking one to two alcoholic beverages on the weekends. He is a truck driver, and he denies any known work exposure. At the time of initial presentation, the patient’s vitals were stable, and on examination, he was well appearing with a soft nondistended abdomen with normoactive bowel sounds. He had significant RUQ pain on palpation with no rebound tenderness and positive Murphy’s sign. He also had occasional bloating and diarrhea, but he denied nausea, vomiting, bloody stools, or chest pain.

Due to the presentation of RUQ pain, radiating to the back, with a history of gallstones, the patient agreed to undergo a robotic-assisted cholecystectomy. Preoperative CT of the abdomen and pelvis with IV contrast as well as preoperative laboratory tests were ordered. The CT revealed a small cyst in the right lobe of the liver with no intrahepatic biliary dilation and no gallbladder wall thickening (Figure 1B). There was an incidental finding of mild airway thickening in the lung bases on the CT, which was correlated to bronchitis; however, no evidence of disease process or other pathological abnormalities was found on the preoperative chest X-ray (Figure 1C, 1D). Basic metabolic panel (BMP), CBC, and glucose level were all within normal limits.

**Figure 2:**
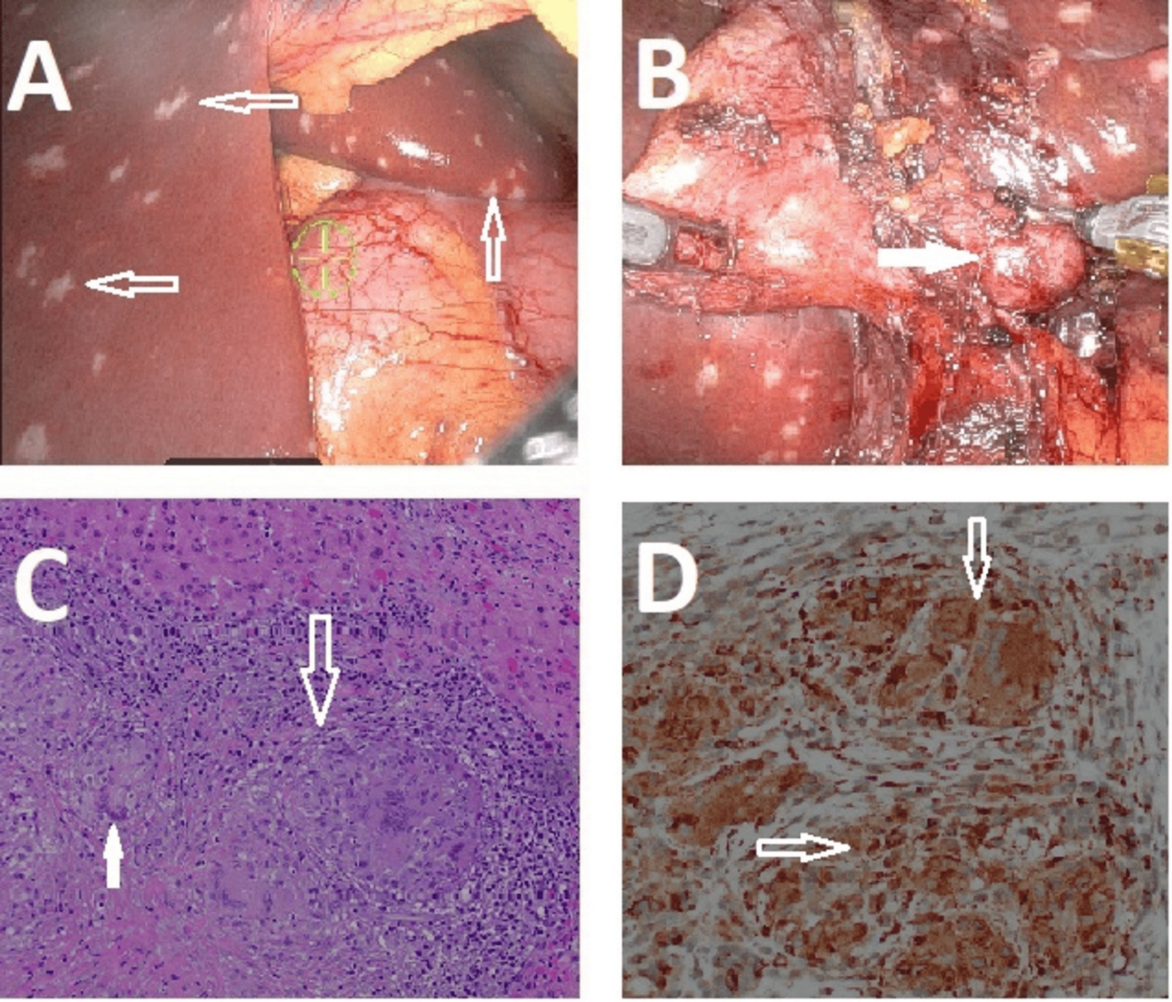
Intraoperative and Histopathological Finding (A) (Intraoperative) Representative intraoperative image of widespread liver lesions (hollow arrows). (B) (Intraoperative) Representative intraoperative image of an enlarged cystic lymph node (hollow arrows). (C) (Histopathology) Representative low-power H&E staining of the liver biopsy specimen tightly packed, confluent non-necrotizing epithelioid granulomas, forming a nodule (hollow arrows). There are multiple giant cells of the Langhans type with nuclei arranged in a horseshoe pattern along the periphery (solid arrows). (D) (Histopathology) Representative high-power immunohistochemical staining showing epithelioid histiocytes and multinucleated giant cells positive for CD68 (hollow arrows). H&E: hematoxylin and eosin

The patient underwent an uneventful assisted cholecystectomy. During the initial inspection of the peritoneal cavity, miliary whitish lesions covering virtually the entire surface of the liver were incidentally discovered (Figure 2A). These lesions were highly suspicious for possible metastatic liver disease from an occult primary tumor, and intraoperative samples were sent to pathology. The surgery proceeded as intended, and the gallbladder was eventually dissected off the liver bed together with a noticeably enlarged lymph node, which also was dissected off (Figure 2B). Due to the suspicious nature of the liver lesions, an intraoperative biopsy of the liver containing one of the suspicious lesions was obtained and sent to pathology as a permanent specimen. The patient returned to the post-anesthesia care unit (PACU) and was discharged home later that day with no complications and in good overall condition.

The pathology report revealed that the liver lesions were consistent with multiple confluent small noncaseating granulomas with giant multinucleated cells present with no malignancy noted on hematoxylin and eosin (H&E) staining (Figure 2C). The enlarged pericystic lymph node was also involved by similar confluent small noncaseating granulomas with histiocytes and giant multinucleated cells that were CD68+ (Figure 2D). There was evident fibrosis with focal hyalinosis. Gallbladder specimen showed chronic acalculous cholecystitis with cholesterolosis and no dysplasia or malignancy.

The patient was seen in the clinic three weeks postoperatively. He was recovering from surgery well without specific complaints and with improved postprandial pain. He was referred to rheumatology for further workup and management.

## Discussion

Sarcoidosis is a multisystem disease presenting as a noncaseating granulomatous reaction typically in the lungs and lymph nodes due to immunological inflammatory reaction [1,6]. Although it can affect all races and ages, the disease is more common among African American and Scandinavian women and typically presents in a bimodal fashion [7]. The etiology of sarcoidosis remains unclear; however, genetic and autoimmune factors have been implicated [3]. The initial presenting symptom of sarcoidosis can be general and vague with fever, fatigue, and cough due to hilar lymphadenopathy [4]. However, many are asymptomatic and are incidentally diagnosed.

The diagnosis of sarcoidosis relies on histopathological evidence of noncaseating granulomas on multiple biopsies and clinical context that excludes other differential diagnoses including malignancy, tuberculosis, and berylliosis when presenting extrapulmonary [7]. On histopathology, the noncaseating granulomas are characteristically formed by tightly bound histocytes in epithelial clusters with multinucleated giant cells surrounded by fibrosis typically in lymph nodes. When sarcoidosis affects the liver, which can be around up to 75% of cases, it usually does so without clinical symptoms or changes in liver function tests and typically does not solely involve the liver [8,9]. Angiotensin-converting enzyme (ACE) levels can be elevated in around 50% of cases, but it is more closely associated with pulmonic disease and can be used as a screening test in those where sarcoidosis may be suspected [8,10].

In this case, the patient’s presentation of chronic cholecystitis led to the incidental diagnosis of sarcoidosis. The preoperative workup showed normal laboratory results with no signs of liver dysfunction, and the preoperative chest X-ray showed no pathological findings. The preoperative CT findings of mild airway thickening in the lung bases are not specific but, given the patient’s longstanding cigarette smoking history, not completely unexpected. However, it could be related to a wide range of possible conditions, including sarcoidosis. An unexpected finding of miliary liver lesions during an unrelated procedure can pose a significant intraoperative concern and diagnostic dilemma, including primary liver conditions such as micronodular cirrhosis, infectious, genetic, immunological, and pharmacological factors, or metastatic disease. As it was in our patient’s case, sarcoidosis can go unnoticed until the final histopathological and immunohistochemical diagnosis has been established. Therefore, biopsy of suspicious lesions should be investigated further in surgical scenarios when unexpected findings present.

Involvement of the liver is an uncommon presentation, but the pathohistological process was also seen in the pericystic lymph node. To date, there have only been a handful of case reports that show sarcoidosis associated with the gallbladder and pericystic lymph node [11]. This rare condition could have at least in part contributed to the patient’s presenting symptoms, especially since cholecystitis is uncommon in young males [12].

Sarcoidosis can affect any organ system and follows a variable course. Many do not require long-term treatment, but approximately 30% may experience chronic disease, necessitating corticosteroid treatment or, as in this case, surgical intervention for gallbladder or lymph node involvement [8,11,13].

## Conclusions

Sarcoidosis is a complex, multisystem disease with diverse and often subtle clinical presentations, making diagnosis challenging, particularly in cases of rare organ involvement. This report highlights an unusual presentation of sarcoidosis involving the gallbladder and pericystic lymph nodes, discovered incidentally during laparoscopic cholecystectomy. Hepatic sarcoidosis is generally uncommon and asymptomatic with absences of significant liver dysfunction.

This case emphasizes the importance of recognizing sarcoidosis as part of the differential diagnosis for atypical intraoperative findings, such as miliary lesions, even in the absence of systemic symptoms or radiographic evidence of pulmonary involvement. A biopsy is necessary to make a diagnosis and should be a part of clinical decision-making if suspicious lesions are encountered. The incidental nature of this diagnosis reinforces the unpredictable course of sarcoidosis and its potential to affect various organ systems silently. Given its rare presentation in the gallbladder and lymph nodes, this case adds to the growing body of literature on extrapulmonary sarcoidosis and highlights the need for further research to guide the clinical management and diagnosis of these uncommon manifestations.

## References

[1] Akalın M, Atıcı SD, Kahraman DS, Tuğmen C (2020). Symptomatic cholelithiasis may be the first sign of sarcoidosis. Rev Assoc Med Bras (1992).

[2] Tajiri T, Hayashi H, Higashi T (2020). Coexisting schwannoma of the gallbladder and sarcoidosis: a case report. Surg Case Rep.

[3] Kumar M, Herrera JL (2019). Sarcoidosis and the liver. Clin Liver Dis.

[4] Flamm SL (2012). Granulomatous liver disease. Clin Liver Dis.

[5] Farman J, Ramirez G, Brunetti J, Tuvia J, Ng C, Rotterdam H (1995). Abdominal manifestations of sarcoidosis. CT appearances. Clin Imaging.

[6] Shah N, Mitra A (2021). Gastrointestinal and hepatic sarcoidosis: a review article. Clin Liver Dis (Hoboken).

[7] Sève P, Pacheco Y, Durupt F (2021). Sarcoidosis: a clinical overview from symptoms to diagnosis. Cells.

[8] Kesici B, Toros AB, Bayraktar L, Dervisoglu A (2014). Sarcoidosis incidentally diagnosed: a case report. Case Rep Pulmonol.

[9] Park YJ, Woo HY, Kim MB, Ahn J, Heo J (2022). Primary hepatic sarcoidosis presenting with cholestatic liver disease and mimicking primary biliary cholangitis: a case report. J Yeungnam Med Sci.

[10] Giovinale M, Fonnesu C, Soriano A (2009). Atypical sarcoidosis: case reports and review of the literature. Eur Rev Med Pharmacol Sci.

[11] Ho JT, Tan HJ, Tan CS, Chiow AK (2019). The odd gallbladder-a rare case of gallbladder and lymph node sarcoidosis: a case report and review of the literature. J Gastrointest Cancer.

[12] Handra-Luca A (2016). Granulomatous lithiasic cholecystitis in sarcoidosis. Clin Pract.

[13] Sollors J, Schlevogt B, Schmidt HJ (2022). Management of hepatic sarcoidosis. J Gastrointestin Liver Dis.

